# Gout

**Published:** 1895-07-06

**Authors:** 


### GENERAL DISEASES.

**Gout.**—Dr. Ralfe[1] considers that too much stress has been laid on uric acid in hypotheses of the pathology of gout. Dr. Ralfe thinks that uric acid is merely a product of a deranged metabolism, this deranged metabolism, in other words gouty paroxysm, being the cause of the uric acid, not the uric acid the cause of the paroxysm. Dr. Ralfe somewhat ridicules the extreme views taken by some as to treatment by diet. He says one prohibits carbo-hydrates, especially sugar, another hydro-carbons, and a third animal food. If the patient were to endeavour to treat himself according to these various theories he would be precluded from any food at all. Dr. Ralfe thinks that the treatment ought to be suited to the individual. In plethoric subjects a wise ascetism, in the feeble some stimulating diet should be adopted. He advocates, both as regards food and alcohol, the dictum of Sydenham, namely, "moderation." The abuse of alcohol is injurious, but complete abstention is worse. Dr. Ralfe decries the indiscriminate use of alkalis, the outcome of hypothetical chemical theories, but thinks that it is safe to carefully attend to the digestion and the condition of the bowels. Improvement of the general health is the main indication. Moderate exercises, such as riding or driving, restore circulation to enfeebled parts. Dr. Ralfe, however, considers Carlsbad waters useful for the plethoric, and those of Homburg for the more feeble. While Dr. Ralfe somewhat abuses the old methods of treatment, Dr. Mordhurst[2] sums up the various reasons for the beneficial action of alkalis in gout. Amongst others may be mentioned: The more alkaline the fluids of the body are the more oxygen they can absorb and retain, the more active is the decomposition of the albuminates, and the greater the quantity of uric acid decomposed. Sodium bicarbonate and sodium chloride are the most useful salts. Sodium chloride promotes digestion, stimulates metabolism, accelerates the flow of lymph, favours the absorption of fluid in the joints, and increases the solvent power of any fluid for uric acid. Sodium bicarbonate and sodium chloride are best tolerated in the form of mineral waters containing small proportions of carbonic acid. Dr. Mordhurst thinks the Wiesbaden waters the best, and that the waters of Vichy and Vals should not be used too long on account of the carbonate of lime they contain. In addition to the use of mineral waters, hot baths and the consequent perspiration in bed have some effect in diminishing the acidity of fluids of the body. Dr. Mapother[3] also writes upon the treatment of gout. Dr. Mapother considers piperazine useful, but also refers to the value of waters containing sodium chloride in solution. He says that the immunity of sailors from gout has been attributed to the quantity of common salt in their food, and on the other hand the frequency of calculous diseases in India is probably due to the small amount of salt taken. Dr. Mapother thinks frequent small meals advisable, since by that means the urine is kept alkaline the greater part of the day.

¹ Lancet, Nov. 10th, 1894. ² The Monatsh, 1894, viii., p. 450; Amer. Med. and Surg. Bulletin, Nov. 1894. ³ Practitioner, Oct. 1894.

